# Molecular Mechanisms of the Ubiquitin-Specific Proteases (USPs) Family in Biliary Tract Cancer and Targeted Intervention Strategies

**DOI:** 10.3390/biomedicines13112586

**Published:** 2025-10-23

**Authors:** Qian Cheng, Delin Ma, Shengmin Zheng, Jialing Hao, Gang Wang, Yanbin Ni, Jiye Zhu

**Affiliations:** 1 Department of Hepatobiliary Surgery, Peking University People’s Hospital, Beijing 100044, China; chengqian@bjmu.edu.cn (Q.C.); 2211110415@stu.pku.edu.cn (D.M.); pkuphzsm@126.com (S.Z.); jialinghaoo@163.com (J.H.); dr_wanggang@bjmu.edu.cn (G.W.); ycnyb@bjmu.edu.cn (Y.N.); 2 Peking University Institute of Organ Transplantation, Peking University, Beijing 100044, China; 3 Beijing Key Laboratory of HCC and Liver Cirrhosis, Peking University People’s Hospital, Beijing 100044, China; 4 Peking University Center of Liver Cancer Diagnosis and Treatment, Peking University People’s Hospital, Beijing 100044, China

**Keywords:** biliary tract cancer, ubiquitin-specific proteases, USP1, USP3, USP7, USP8, USP9X, USP21, USP22

## Abstract

Biliary tract carcinoma (BTC) is a group of highly heterogeneous malignancies arising from the biliary epithelium. Anatomically, BTC is categorized into gallbladder cancer (GBC) and cholangiocarcinoma (CCA), with the latter further subdivided into intrahepatic (iCCA), perihilar (pCCA), and distal cholangiocarcinoma (dCCA). Epidemiological studies reveal a dismal five-year survival rate of less than 20% for BTC patients, with limited responses to current chemotherapy regimens, underscoring the urgent need to unravel its complex molecular pathogenesis. Recent research has increasingly focused on the regulatory networks of post-translational modifications, particularly the ubiquitin-proteasome system (UPS), in tumorigenesis. As the largest subfamily of deubiquitinating enzymes (DUBs), ubiquitin-specific proteases (USPs) regulate the stability of key oncoproteins such as phosphatase and tensin homolog (PTEN) and c-Myc, playing pivotal roles in tumor cell proliferation, apoptosis evasion, invasion, and metastasis. This review systematically summarizes the differential expression profiles of USP family members (e.g., USP1, USP3, USP7, USP8, USP9X, USP21, and USP22) in BTC and their clinical significance, with a focus on elucidating how specific USPs regulate tumor progression through key substrates, including poly(ADP-ribose) polymerase 1 (PARP1), dynamin-1-like protein (DNM1L), and O-GlcNAc transferase (OGT). Furthermore, based on recent advances, we discuss the therapeutic potential of small-molecule USP inhibitors in BTC targeted therapy, providing a theoretical foundation for developing novel precision treatment strategies.

## 1. Introduction

Biliary tract cancer (BTC) is an umbrella term referring to a group of heterogeneous and aggressive adenocarcinomas, including cholangiocarcinoma (CCA) and gallbladder cancer (GBC) [1,2]. Based on anatomical location, CCA is further classified into intrahepatic cholangiocarcinoma (iCCA), perihilar cholangiocarcinoma (pCCA), and distal cholangiocarcinoma (dCCA), originating from the intrahepatic bile ducts, perihilar bile ducts, and extrahepatic distal bile ducts, respectively. Biliary tract cancers are often insidious in onset, with most patients diagnosed at an advanced stage due to the lack of early symptoms, resulting in a poor prognosis. In recent years, the incidence of CCA has been on the rise [2]. However, due to the lack of apparent symptoms in the early stages, over 70% of patients are diagnosed at an advanced stage, with a 5-year survival rate of only 7–20% [3,4,5]. In addition, GBC has a five-year survival rate of less than 20%, highlighting the challenges in treatment [6]. Currently, radical surgical resection remains the primary treatment for biliary tract tumors, yet therapeutic outcomes for advanced-stage patients remain unsatisfactory. Therefore, further research into the pathogenesis of biliary tract cancers and their molecular pathological characteristics is crucial for developing novel diagnostic and therapeutic strategies, ultimately improving patient survival and prognosis.

Protein ubiquitination is a post-translational modification driven by a cascade reaction mediated by ubiquitin-activating enzymes (E1), ubiquitin-conjugating enzymes (E2), and ubiquitin-protein ligases (E3) [7,8]. Deubiquitinating enzymes (DUBs) can recognize ubiquitinated proteins and hydrolytically remove ubiquitin molecules from substrate proteins, thereby achieving dynamic and reversible regulation of protein ubiquitination [9]. The ubiquitination enzyme system and DUBs together form a complex ubiquitin regulatory network that is involved in the multidimensional modulation of cellular life activities [10]. Recent studies have highlighted the critical involvement of post-translational modifications, particularly ubiquitination and deubiquitination, in the pathogenesis of biliary tract cancer. Among DUBs, the ubiquitin-specific protease (USP) family has emerged as a key regulator of oncogenic signaling, tumor microenvironment remodeling, and therapeutic resistance in BTC.

This article systematically reviews the multi-dimensional regulatory roles of the ubiquitin-specific proteases (USPs) family in the occurrence and development of biliary tract tumors, with a focus on elucidating the molecular mechanisms by which this protein family affects key oncogenic signaling pathways through deubiquitination. Through in-depth analysis of the downstream signaling networks mediated by USPs (including but not limited to the Wingless-type (Wnt)/β-catenin, phosphatidylinositol 3-kinase/protein kinase B (PI3K/AKT), and mitogen-activated protein kinase (MAPK) pathways), we have revealed their central regulatory positions in the proliferation, metastasis, and drug resistance formation of biliary tumor cells. Based on existing research evidence, this article further explores the development strategies and clinical application prospects of small molecule inhibitors and proteolysis targeting chimera (PROTAC) degraders targeting members of the USPs family. This review aims to provide a new perspective for a deeper understanding of the post-translational regulation of biliary tract tumors and expects to inspire more scholars to explore emerging research directions, such as the interaction of the USP family with the biliary tumor microenvironment and metabolic reprogramming, ultimately providing a theoretical basis for the development of precise treatment strategies.

## 2. The Ubiquitin-Proteasome System (UPS) and USPs

### 2.1. UPS

UPS is the principal route for selective protein degradation in eukaryotes. By eliminating misfolded, damaged, or short-lived regulatory proteins, UPS preserves proteostasis and orchestrates cell-cycle progression, signal transduction, transcription, DNA repair, and immune responses. Its core components are ubiquitin, E1-activating enzymes, E2-conjugating enzymes, E3 ligases, the 30S proteasome, and deubiquitinating enzymes (DUBs).

Ubiquitin is a small protein composed of 76 amino acids (molecular weight 8.5 kDa) that contains seven lysine residues (K6, K11, K27, K29, K33, K48, and K63), which can form ubiquitin chains [11]. As a “molecular tag”, ubiquitin regulates protein fate through covalent attachment to target proteins (typically via the ε-amino group of lysine residues on substrate proteins). Depending on the modification pattern, ubiquitination can be classified into monoubiquitination, multi-monoubiquitination (multiple-site monoubiquitination), or polyubiquitination [12]. The latter is further subdivided into homotypic (uniform linkage) and heterotypic (mixed linkage) chains. Different ubiquitin linkage types mediate distinct regulatory functions: for example, K48-linked polyubiquitin chains primarily serve as signals for proteasome-dependent protein degradation, while other linkages (e.g., K63) are involved in non-degradative processes such as signaling modulation [7,12].

The ubiquitin-enzyme system catalyzes protein ubiquitination by covalently attaching ubiquitin molecules to substrate proteins, thereby regulating protein degradation, subcellular localization, activity, and interactions. This process is executed cooperatively by three enzymes: the E1, E2, and E3 [12]. In an ATP-dependent manner, E1 activates ubiquitin to form an E1~ubiquitin thioester and transfers the activated ubiquitin to E2. Upon receiving ubiquitin, E2—together with E3—determines the linkage topology of the ubiquitin chains (e.g., K48- or K63-linked). E3 specifically recognizes substrate proteins and catalyzes the transfer of ubiquitin from E2 to the substrate. Its substrate selectivity defines the specificity of ubiquitination, making E3 the pivotal component of the regulatory network.

As one of the most important and intricate holoenzyme machines in the cell, the human 30S proteasome comprises about 66 distinct subunits and has a total atomic mass of approximately 2.5 MDa [13]. Structurally, the 30S (subunit) proteasome is assembled from the 20S core particle, capped on each end by a 19S regulatory particle. The 19S regulatory particle recognizes, unfolds, and translocates ubiquitin-tagged protein substrates into the 20S core particle for degradation [13]. The 20S core particle is composed of an outer α-ring and an inner β-ring; the β-ring houses the catalytic chamber, in which the β1, β2, and β5 subunits harbor caspase-like, trypsin-like, and chymotrypsin-like active sites, respectively, responsible for proteolytic activity [13].

Ubiquitination is a tightly regulated and reversible process. DUBs reverse ubiquitin modifications by hydrolyzing peptide or isopeptide bonds between ubiquitin molecules or between ubiquitin and substrate proteins [14]. DUBs not only suppress ubiquitin-related processes but also facilitate them by disassembling ubiquitin chains, recycling free ubiquitin molecules, and correcting erroneous ubiquitination [15]. Together with the ubiquitin system, they form a sophisticated and dynamic regulatory network [15]. Among the 500 genes encoding proteases in the human genome, approximately 20% are involved in encoding over 100 DUBs [16]. DUBs precisely regulate ubiquitin signaling dynamics by hydrolyzing ubiquitin chains or removing ubiquitin modifications from substrate proteins [16]. Based on catalytic domain sequence conservation, enzymatic mechanisms, and structural features, DUBs are classified into nine superfamilies as follows: USPs, ovarian tumor proteases (OTUs), ubiquitin C-terminal hydrolases (UCHs), Josephins (MJDs, Machado–Joseph Disease Proteases), JAMM/MPN^+^metalloproteases (JAMMs), MIU-containing novel DUB family (MINDYs), monocyte chemotactic protein-induced proteins (MCPIPs), Papain-like peptidases of dsRNA viruses and eukaryotes (PPPDEs), and zinc finger-containing ubiquitin peptidase 1 (ZUP1) [16]. DUB dysregulation is implicated in cancer (e.g., USP28 stabilizes MYC oncoprotein), neurodegenerative disorders (e.g., UCH-L1 loss-of-function), and autoinflammatory diseases (e.g., OTULIN deficiency), making them promising therapeutic targets [17,18,19,20,21].

### 2.2. USPs

The USP family belongs to cysteine proteases and represents the largest and most structurally diverse subclass of DUBs, comprising over 50 members that account for approximately 60% of all human DUBs [22]. The molecular masses of USPs range from approximately 50 to 300 kDa. As cysteine-dependent proteases, USP enzymes share a catalytic mechanism analogous to papain, featuring a catalytic domain composed of three highly conserved subdomains that adopt a characteristic “finger-thumb-palm” topological fold [23,24]. The hallmark of USP family members is the conserved USP domain, which confers protease activity to hydrolyze ubiquitin-substrate conjugates. Beyond the core catalytic domain, most USPs contain auxiliary structural modules such as ubiquitin-like domains (UBL), zinc finger ubiquitin-binding domains (ZnF-UBP), USP-specific domains (DUSP), ubiquitin-interacting motifs (UIM), and ubiquitin-associated domains (UBA) [23]. These accessory domains expand functional versatility by modulating substrate recognition, protein–protein interaction networks, and subcellular localization. Notably, USPs exhibit remarkable substrate selectivity, enabling precise regulation of specific ubiquitinated targets to influence diverse signaling pathways and cellular processes [23]. Compartmentalization represents another key feature—some USPs predominantly localize to the nucleus while others reside in the cytoplasm, providing spatial control over deubiquitination events [24]. USPs play crucial roles in a variety of cellular functions, including cell cycle regulation, DNA damage repair, gene expression regulation, and the cellular signaling axis [25,26].

## 3. USPs and CCA

Recent studies have demonstrated that multiple USP family members exhibit dysregulated expression in biliary tract malignancies—including GBC and CCA—and directly contribute to tumorigenesis, progression, and clinical outcomes through modulation of key oncogenic signaling pathways.

### 3.1. USP1

#### 3.1.1. Molecular Characteristics and Functions of USP1

**Figure 1:**
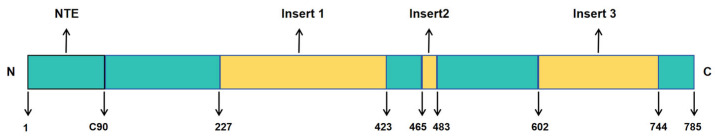
Schematic representation of the USP1 primary structure. The catalytic core comprises a conserved N-terminal Cys box and a C-terminal His box that contains His593 and Asp751. The enzyme also contains three insertion segments: insert 1 (L1), insert 2 (L2), and insert 3 (L3).

USP1 is one of the most extensively studied DUBs, encoded by the USP1 gene, comprising 785 amino acids with a relative molecular mass of 88.2 kDa [27]. USP1 contains a canonical USP-type deubiquitinase (DUB) domain that is highly conserved across the family. This domain comprises an N-terminal Cys-box, in which C90 serves as the catalytic cysteine, and a C-terminal His-box containing the catalytic histidine H593 and the catalytic aspartate D751 (Figure 1) [27]. Compared to its paralogs, USP1 possesses one of the largest catalytic domains within the USP family, primarily due to three insertion segments within its conserved catalytic core [28]. The first insertion, L1 (residues 227–423), serves as a central hub for allosteric regulation of USP1 activity. It contains phosphorylation sites (e.g., S313), nuclear localization signals (NLSs), and a degradation motif (degron). The second insertion, L2 (residues 602–744), harbors an autocleavage site (G670–G671), while the third, L3 (residues 465–483), collaborates with L1 to mediate autoinhibition. Binding of the cofactor UAF1 (USP1-associated factor 1) reverses this inhibition, and concurrent deletions of L1 and L3 lead to hyperactivation of USP1 (Figure 1) [29,30].

USP1 is involved in diverse cellular biological processes such as DNA damage response, immune regulation, cell proliferation, apoptosis, and migration [30]. Its expression and activity are finely regulated by multiple mechanisms, including transcriptional regulation, phosphorylation, autocleavage, and proteasomal degradation, ensuring that USP1 functions in a spatiotemporally appropriate manner within cells [30]. Moreover, dysregulated expression and activity of USP1 have been observed in various human cancers, suggesting its feasibility as a therapeutic target for anticancer treatment [30].

#### 3.1.2. Role and Molecular Mechanisms of USP1 Family in CCA

Zhang et al. (2023) demonstrated that USP1 is markedly upregulated in CCA tissues compared with adjacent normal tissues, and that elevated USP1 promotes CCA proliferation and metastasis [31]. Using proteomic profiling, proximity-labeling assays, and glutathione S-transferase (GST) pull-down experiments, they verified a direct interaction between USP1 and poly (ADP-ribose) polymerase 1 (PARP1) that occurs predominantly within the nucleus. Notably, this USP1–PARP1 association is independent of USP’s deubiquitinase activity. Further mapping revealed that the amino acid segment 401–785 of USP1 constitutes the critical region required for binding PARP1 and the amino acid segment 203–476 of PARP1, which includes the BRCT domain, is responsible for interacting with USP1. USP1 removes the K48-linked ubiquitin chain from PARP1-K197, thereby preventing proteasomal degradation; the K197R mutant cannot be stabilized by USP1. In summary, USP1 stabilizes the PARP1 protein in CCA by deubiquitinating PARP1-K197 and thereby blocking its proteasomal degradation.

In addition to ubiquitination, acetylation is a critical post-translational modification that targets lysine residues and plays a pivotal role in regulating diverse cellular processes, including protein–protein interactions, transcriptional regulation, subcellular localization, and enzymatic activity. Acetylation regulates protein stability through two key mechanisms: (1) by competing with ubiquitination for occupancy at the same lysine site, and (2) by modulating the association or dissociation of protein complexes with E3 ubiquitin ligases or deubiquitinases (DUBs), thereby promoting either protein degradation or stabilization. Through functional experiments, the authors identified General Control of Amino Acid Synthesis 5-Like 2 (GCN5, also known as Lysine Acetyltransferase 2A, KAT2A) as the primary acetyltransferase responsible for USP1 acetylation, which enhances USP1’s deubiquitinating activity toward PARP1. Mechanistic studies demonstrated that acetylation at lysine 130 (K130) of USP1 is both necessary and sufficient for this regulatory effect, as evidenced by experiments using non-acetylatable and acetylation-mimetic mutants. Notably, although prior studies identified karyopherin subunit alpha 2 (KPNA2, in breast cancer metastasis) and ribosomal protein S16 (RPS16, in hepatocellular carcinoma) as USP1 substrates, these proteins were not detected as USP1 targets in the current CCA model. This finding suggests that USP1 may exhibit context-dependent functional specificity and substrate preferences across different tumor types [32,33].

To evaluate the clinical significance of the USP1–PARP1 axis in CCA, the researchers systematically analyzed the correlation between USP1 and PARP1 protein expression in human CCA tissues. Western blot analysis of 28 CCA specimens revealed a significant positive correlation between USP1 and PARP1 protein levels. Further validation with an expanded cohort of 65 cases by immunohistochemical staining consistently demonstrated a significant positive correlation in protein expression. Kaplan–Meier survival analysis (*n* = 65) showed that high expression of either USP1 or PARP1 was significantly associated with shorter overall survival. These findings suggest that USP1 plays a critical role in CCA progression by regulating PARP1 protein levels, and both proteins may serve as independent molecular biomarkers for predicting poor prognosis in CCA patients.

**Figure 2:**
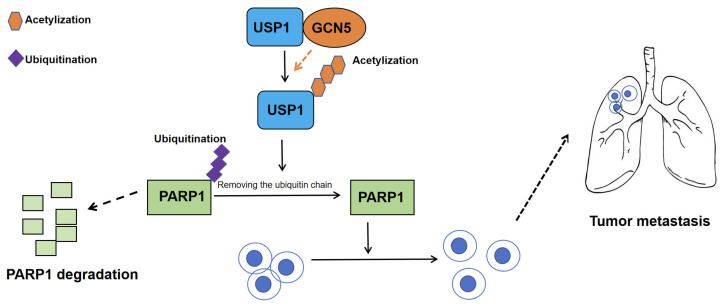
The USP1–PARP1 regulatory axis in CCA. Schematic model illustrating the GCN5-USP1-PARP1 signaling cascade. GCN5-mediated acetylation of USP1 at K130 enhances its binding to PARP1, promoting the removal of K48-linked ubiquitin chains from PARP1 at K197. This deubiquitination stabilizes PARP1 by preventing its proteasomal degradation, ultimately driving CCA progression.

In summary, this study is the first to elucidate the tumor-promoting role of the USP1–PARP1 axis in cholangiocarcinoma, filling a critical gap in the understanding of USP1 in this malignancy (Figure 2). Additionally, it unveils the “GCN5-USP1-PARP1” acetylation–deubiquitination cascade regulatory network, providing a novel theoretical framework for understanding how protein homeostasis imbalance drives malignant tumor progression (Figure 2). Given that PARP1 inhibitors have already received FDA approval for treating DNA damage repair-deficient tumors, this study further establishes a solid experimental and theoretical foundation for extending the application of USP1 inhibitors, GCN5 inhibitors, or PARP1 inhibitors to cholangiocarcinoma, potentially paving the way for new combination targeted therapies. The protein expression levels of USP1 and PARP1, as well as the acetylation status of USP1-K130, could serve as potential prognostic biomarkers to guide precision stratification therapy in cholangiocarcinoma.

### 3.2. USP8

#### 3.2.1. Molecular Characteristics and Molecular Functions of USP8

**Figure 3:**
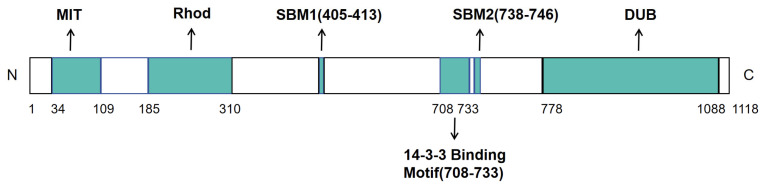
Modular organization of USP8. Linear domain architecture of USP8 with amino acid positions: MIT (34–109), Rhod (185–310), SBM1 (405–413), SBM2 (738–746), 14-3-3 binding motif (708–733), and catalytic core (778–1088). The catalytic triad is formed by C786/H1067/N1088.

USP8 (Ubiquitin-specific protease 8) is a modular deubiquitinating enzyme composed of 1118 amino acids, characterized by a classic tri-domain architecture (Figure 3) [34]. The USP8 protein contains five functionally distinct segments: the microtubule-interacting and trafficking (MIT) domain (residues 34–109), the Rhodanese-like (Rhod) domain (residues 185–310), two SH3-binding motifs (SBMs) (residues 405–413 and 738–746), 14-3-3-binding motif (14-3-3-BM) (residues 708–733), and the deubiquitinase catalytic (DUB) domain (residues 778–1088) (Figure 3) [34]. The N-terminal segment (residues 1–313) contains two key regulatory domains: the MIT (Microtubule-interacting and transport) domain mediates binding to the endosomal sorting complexes required for transport III (ESCRT-III) complex for membrane protein sorting, while the adjacent Rhod (Rhodanese homology) domain, whose function remains incompletely understood, may participate in sulfurtransferase activity. The central region (314–714 aa) harbors sophisticated regulatory elements, including an SH3-binding site, a WW-like autoinhibitory domain (645–684 aa), and multiple phosphorylation sites (e.g., Ser680/Ser718) that can be modified by kinases and bind 14-3-3 proteins to modulate enzymatic activity through conformational changes. The C-terminal catalytic core (715–1118 aa) forms the canonical USP-family fold, featuring a conserved catalytic triad (Cys786-His1067-Asn1088) that specifically recognizes and cleaves K27- and K48-linked ubiquitin chains. This catalytic domain contains a ubiquitin-binding groove and allosteric regulatory sites, with the C-terminal tail (1100–1118 aa) potentially further modulating substrate recognition. This multi-domain organization enables USP8 to integrate diverse signals (e.g., phosphorylation, protein–protein interactions) for precise spatiotemporal regulation of its deubiquitinating activity.

USP8 stabilizes a broad range of substrates—including programmed death-ligand 1 (PD-L1), epidermal growth factor receptor (EGFR), melanoma differentiation-associated protein 5 (MDA5), charged multivesicular body protein 1B (CHMP1B), and beta-secretase 1 (BACE1)—thereby regulating critical cellular processes such as receptor recycling, autophagy, mitophagy, innate immunity, and anti-tumor immune responses [35,36,37,38,39,40]. Dysregulation of USP8 contributes to disease pathogenesis through its deubiquitinase activity in multiple pathways. Hot-spot mutations in USP8—such as S718P and S719del—disrupt 14-3-3-mediated autoinhibition in corticotroph adenomas, leading to sustained stabilization of EGFR and hyperactivation of downstream MAPK and PI3K-AKT signaling, ultimately causing Cushing’s disease [36]. In solid tumors, including gastric, hepatocellular, cholangiocarcinoma, and pancreatic cancer, elevated USP8 expression suppresses anti-tumor immunity and drives tumor proliferation, invasion, and resistance to targeted therapies [35,41,42,43,44,45]. Moreover, aberrant deubiquitination of α-synuclein, tau, and amyloid precursor protein (APP) by USP8 is intimately linked to the onset and progression of neurodegenerative disorders such as Parkinson’s and Alzheimer’s diseases [39,40]. Consequently, USP8 is emerging as a promising therapeutic target in oncology, endocrinology, and neurology.

#### 3.2.2. USP8 Stabilizes O-GlcNAc Transferase (OGT) via Deubiquitination to Drive iCCA Progression and Pemigatinib Resistance

Elevated USP8 expression has been identified in various digestive system tumors, including gastric cancer, hepatocellular carcinoma (HCC), cholangiocarcinoma, and pancreatic cancer [35,41,42,43,44,45]. In these malignancies, elevated USP8 levels are associated with tumor progression, metastatic potential, higher recurrence rates, and unfavorable clinical outcomes. By cleaving K48-linked ubiquitin chains on β-catenin, the deubiquitinase USP8 prevents its degradation and enhances nuclear accumulation, thereby sustaining Wnt/β-catenin signaling. This drives hepatocellular proliferation and invasion and confers ferroptosis resistance through up-regulation of c-Myc, Cyclin D1 and glutathione peroxidase 4 (GPX4), fueling the malignant progression of hepatocellular carcinoma [42,43]. Yang et al. discovered that the deubiquitinase USP8 directly removes ubiquitin chains from PD-L1 [35]. Compared with normal tissues, pancreatic cancer tissues exhibit significantly elevated USP8 expression, and across multiple patient cohorts, USP8 levels positively correlate with tumor-node-metastasis (TNM) staging. Functionally, USP8 depletion suppresses tumor invasion, migration, and volume in an immune system-dependent manner, while enhancing anti-tumor immunogenicity. Pretreatment with USP8 inhibitors reduces tumorigenesis, and USP8-knockdown tumor models in immunocompetent mice demonstrate significantly prolonged survival. Mechanistically, USP8 directly interacts with PD-L1 and upregulates PD-L1 protein levels by blocking the ubiquitin-proteasome degradation pathway. Combined treatment with a USP8 inhibitor and anti-PD-L1 therapy markedly inhibits pancreatic tumor growth, mediated through activation of cytotoxic T cells and the PD-L1/CD8^+^ T-cell axis. Collectively, targeting USP8 sensitizes tumors to PD-L1-targeted immunotherapy, offering a novel therapeutic strategy for pancreatic cancer patients [35].

In 2024, Long et al. published groundbreaking research in Cancer Cell International elucidating the pivotal role of the deubiquitinase USP8 in iCCA progression [44]. Their work demonstrated that USP8 significantly promotes iCCA proliferation and invasion. The study pioneered the discovery that USP8 governs O-GlcNAc transferase (OGT) ubiquitination, a previously unrecognized regulatory axis. Co-immunoprecipitation (Co-IP) confirmed direct USP8-OGT interaction, with USP8 promoting the stability of OGT protein levels. Knockdown of USP8 markedly decreased OGT expression, while reconstitution with wild-type USP8 (USP8-WT) restored OGT levels. Crucially, USP8 depletion failed to further reduce OGT stability upon proteasome inhibition by MG132, and shortened OGT’s half-life, collectively proving that USP8 sustains OGT stability via deubiquitination to fuel iCCA pathogenesis.

While USP8 was known to cleave K6-, K48-, and K63-linked ubiquitin chains, this study revealed its dose-dependent removal of ubiquitin moieties from OGT. Systematic ubiquitin mutagenesis (K6, K11, K27, K29, K33, K48, and K63) identified K27- and K48-linked chains as USP8’s specific targets on OGT, thereby blocking OGT degradation via the proteasomal pathway. Notably, K48-linked polyubiquitination typically marks proteins for proteasomal degradation, whereas K27-linked ubiquitination—whose physiological roles remain enigmatic—has recently been implicated in autoimmunity and DNA damage repair.

Rescue experiments confirmed that OGT overexpression reversed the anti-tumor effects of USP8 knockdown, solidifying the USP8–OGT axis as a core driver of iCCA. O-GlcNAcylation, the PTM catalyzed by OGT and reversed by O-GlcNAcase (OGA), plays established roles in tumorigenesis. Prior studies linked OGT to cancer metastasis via autophagy and ferroptosis regulation, suggesting O-GlcNAcylation modulation as a therapeutic avenue.

**Figure 4:**
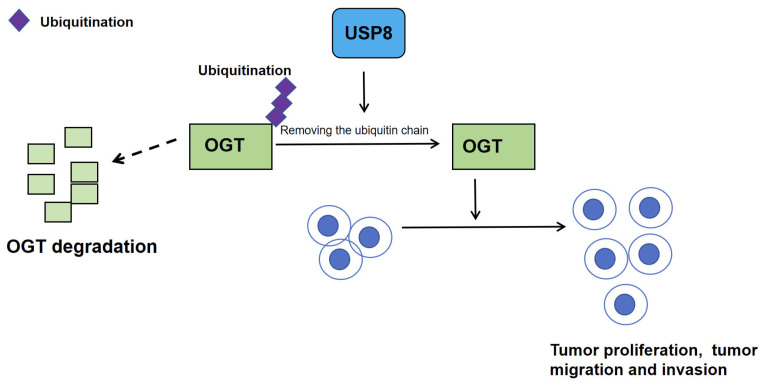
USP8-mediated stabilization of OGT drives iCCA progression. Graphical summary of the USP8–OGT axis. USP8 deubiquitylates OGT by removing K27- and K48-linked ubiquitin chains, thereby preventing its proteasomal degradation. Stabilized OGT enhances global O-GlcNAcylation and accelerates tumor proliferation.

In conclusion, the aforementioned study unveils USP8 as the deubiquitinase stabilizing OGT to promote iCCA aggressiveness (Figure 4) [44]. These findings deepen the mechanistic understanding of iCCA and provide a translational framework for combination therapies. Targeting the USP8–OGT axis may emerge as a promising strategy in precision oncology for iCCA.

### 3.3. USP9X

#### 3.3.1. Molecular Characteristics and Functions of USP9X

**Figure 5:**
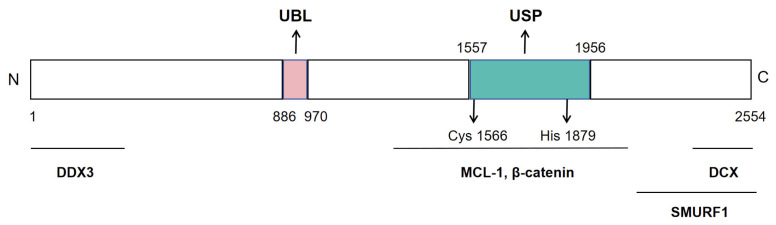
Primary structure of USP9X. Linear map indicating the UBL domain (aa 886–970), the catalytic triad (C1566/H1879/D1882), and the zinc-finger motif (Cys-X-X-Cys).

USP9X is a 2550-amino-acid X-linked protein that adopts an elongated monomeric architecture composed of three hierarchical levels: (i) residues 886–970 form an N-terminal ubiquitin-like domain (UBL) that mediates protein–protein interactions and subcellular localization; (ii) the catalytic core (1557–1956) folds into the canonical USP “finger-palm-thumb” scaffold, which is interspersed by a unique β-hairpin insertion that confers additional conformational flexibility; and (iii) the catalytic triad Cys1566-His1879-Asp1882 sits between the finger and palm subdomains, flanked by a Zn^2+^-binding zinc-finger motif (Cys-X-X-Cys) and three tandem ubiquitin-binding pockets that collectively dictate cleavage specificity toward K11 > K63 > K48 > K6 ubiquitin linkages (Figure 5) [46]. This structural model has been validated by both 2.5 Å crystallographic data and AlphaFold homology modeling, with pathogenic mutations in the zinc-finger region (e.g., Glu1764Lys, Asp1761Tyr) shown to impair zinc coordination or spatial conformation, thereby attenuating enzymatic activity and ubiquitin-chain recognition [46].

USP9X uses its characteristic “finger–palm–thumb” catalytic cleft to recognize and cleave K11-, K48-, and K63-linked polyubiquitin chains from substrates, thereby preventing proteasomal degradation or modulating signal-complex assembly. The enzyme is essential for cell-cycle progression, DNA-damage repair, and transforming growth factor-beta (TGF-β)/SMA/mothers against decapentaplegic homolog (Smad), Wnt/β-catenin, and Notch signaling by stabilizing key proteins such as myeloid cell leukemia-1 (MCL-1), axis inhibition protein (AXIN), SMAD4, and Itchy E3 ubiquitin protein ligase (ITCH), thereby controlling apoptosis, stem-cell maintenance, and epithelial–mesenchymal transition; loss-of-function or mutations in USP9X lead to developmental defects and increased susceptibility to various cancers [46].

#### 3.3.2. USP9X Regulates Apoptosis in Cholangiocarcinoma

In 2021, Chen et al. elucidated the mechanism by which USP9X regulates apoptosis in CCA [47]. Their study revealed that USP9X suppresses the malignant potential of CCA by modulating the prolyl 4-hydroxylase domain protein 3 (EGLN3). Specifically, USP9X stabilizes EGLN3 protein through deubiquitination, leading to the upregulation of apoptosis-related proteins such as kinesin family member 1B Beta isoform(KIF1Bβ). This process promotes cancer cell apoptosis and inhibits tumor progression, positioning the USP9X/EGLN3 axis as a promising diagnostic and therapeutic target for CCA.

Ubiquitination dynamically regulates protein stability and turnover, a process orchestrated by ubiquitinating enzymes and deubiquitinases (DUBs). The human genome encodes nearly 100 DUBs, many of which—including USP9X—exhibit aberrant expression in cancer. Intriguingly, USP9X plays a context-dependent role across cancer types and stages. For instance, in lung cancer, USP9X drives tumor progression by stabilizing the oncoprotein lysine-specific demethylase 4C (KDM4C). In contrast, in pancreatic cancer, it suppresses tumorigenesis by maintaining large tumor suppressor kinase 2 (LATS2) stability in the Hippo pathway. This study further demonstrates USP9X’s tumor-suppressive function in CCA, mediated through EGLN3 stabilization.

EGLN3, a member of the EGLN family, functions as a prolyl hydroxylase and has been implicated as a tumor suppressor in multiple cancers. Research indicates that EGLN3 inhibits tumor growth by suppressing EGFR signaling, inducing G1-phase cell cycle arrest, and promoting apoptosis [47]. In this study, the authors uncovered that USP9X enhances EGLN3 protein stability via deubiquitination, thereby reinforcing its tumor-suppressive effects.

**Figure 6:**
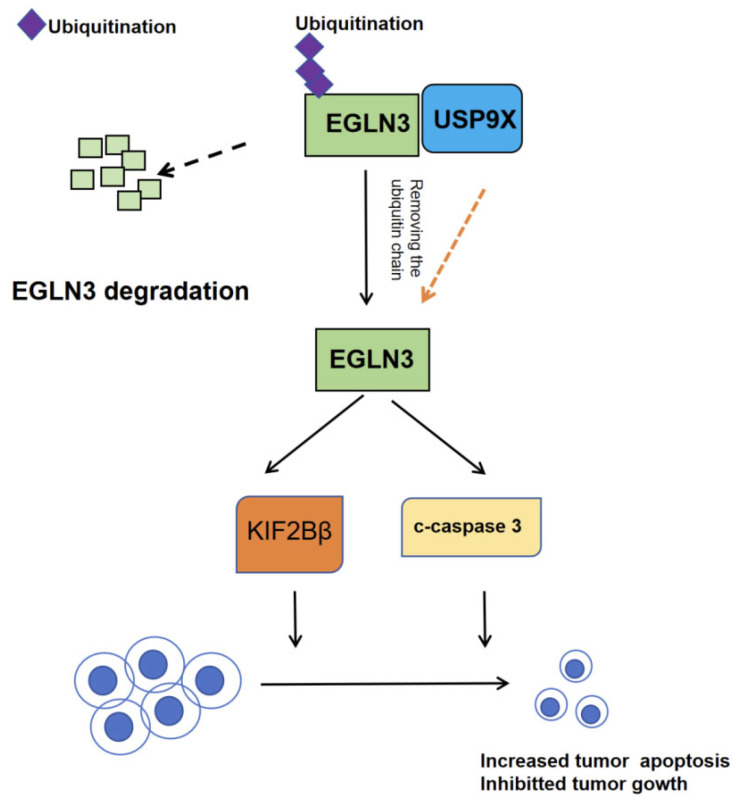
Tumor-suppressive role of USP9X in CCA progression. USP9X functions as a tumor suppressor by stabilizing EGLN3 through deubiquitination. Genetic depletion of EGLN3 completely reverses the anti-proliferative effects mediated by USP9X. Mechanistically, USP9X upregulates the pro-apoptotic factor KIF1Bβ in an EGLN3-dependent manner, thereby inducing programmed cell death in CCA cells. These findings establish USP9X as both a molecular suppressor of CCA progression and a promising diagnostic/therapeutic target for this malignancy.

In summary, this study is the first to demonstrate that USP9X exerts tumor-suppressive effects in cholangiocarcinoma by deubiquitinating and stabilizing the EGLN3 protein, thereby activating the KIF1Bβ-mediated apoptotic pathway and establishing a novel USP9X-EGLN3-KIF1Bβ tumor-suppressive axis (Figure 6). Comprehensive validation using clinical samples, cell lines, and animal models has established USP9X as a potential prognostic biomarker and therapeutic target for cholangiocarcinoma, challenging the previous notion of USP9X as solely an oncogenic factor and providing both a theoretical basis and new translational directions for precision diagnosis and treatment of this malignancy.

### 3.4. USP21

#### 3.4.1. Molecular Characteristics and Molecular Functions of USP21

**Figure 7:**
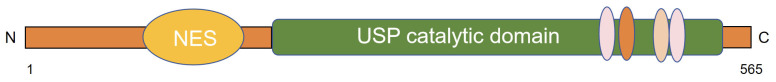
Domain architecture of USP21. Unlike most DUBs, USP21 exhibits a relatively simple structural organization. The N-terminal region (residues 1–212) is intrinsically disordered, while the C-terminal portion harbors the conserved catalytic domain.

The human ubiquitin-specific protease 21 (USP21) gene is located on chromosome 2q36.1, and encodes a 565-amino acid polypeptide. According to ExPASy prediction (https://cn.expasy.org), the theoretical molecular weight of this protein is 62.656 kDa, with an isoelectric point (pI) of 9.89 [48]. Its N-terminal region (residues 1–212) is intrinsically disordered (IDR), while the C-terminus forms a complete catalytic domain. Secondary structure prediction (SOPMA) reveals 26.68% α-helices (151 residues), 3.18% β-bridges (18 residues), 59.01% random coils (334 residues), and 11.13% extended strands (63 residues). Among the 56 reported human USP members, USP21 clusters with USP2, sharing ~50% overall sequence homology, particularly in the catalytic domain (Figure 7). Notably, USP21’s S2 binding site (Arg441-Gln442-Lys443-Thr444) differs from USP2’s (Arg441-Gly442-Arg443-Lys444), providing a structural basis for selective inhibitor design.

The catalytic domain adopts an “open-hand” conformation, comprising thumb, fingers, and palm subdomains. The crystal structure with di-ubiquitin aldehyde (PDB: 2Y5B) shows the catalytic triad (Cys221-His518-Asp534) positioned between the thumb and palm, while the fingers subdomain binds ubiquitin. Six conserved motifs (Box 1–6) are present: Box 1 contains catalytic Cys, Box 5 harbors catalytic His, and Box 6 includes catalytic Asp/Asn. Box 3 and Box 4 feature Cys-X-X-Cys zinc-binding motifs, critical for structural stability and long-range interactions. Chelation or substitution of Zn^2+^ disrupts the motif, destabilizing the protein—highlighting this site as a potential therapeutic target. As a cysteine protease, USP21’s catalytic domain (residues 212–558) mediates deubiquitination via S1 (recognizing ubiquitin’s Ile44 patch), S1′, and S2 sites, enabling chain-type specificity (e.g., K48/K63). The open ubiquitin-binding pocket, zinc-binding motifs, and unique S2 site collectively offer a framework for developing selective inhibitors.

USP21, a pivotal member of the USP subfamily, plays crucial roles in fundamental biological processes, including apoptosis, DNA repair, proliferation regulation, and signal transduction. This deubiquitinating enzyme exhibits broad tissue distribution, with significant expression in fallopian tubes, cerebellum, seminal vesicles, and other organs. As a C19 peptidase family member, USP21 demonstrates remarkable specificity in recognizing and cleaving K48- and K63-linked ubiquitin chains, thereby precisely modulating protein degradation pathways.

Mechanistically, USP21 orchestrates protein homeostasis through dual regulatory mechanisms: mediating K48-chain deubiquitination to influence proteasome-dependent degradation, and catalyzing K63-chain removal to modulate signal transduction, kinase activation, and autophagy processes. For instance, USP21 directly interacts with FOXD1 (Forkhead box D1) to promote tumorigenesis by eliminating its ubiquitin modifications [49]. USP21 efficiently disassembled K48-linked polyubiquitylation of FOXD1 but had no significant effect on monoubiquitylation or the K11-, K27-, and K63-linked polyubiquitylation of FOXD1 [49]. USP21 inhibits inflammatory responses and cell survival signaling by removing K63-linked ubiquitin chains from receptor-interacting protein 1 (RIP1), thereby blocking RIP1-mediated NF-κB activation [50]. Additionally, USP21 cleaves both K27- and K63-linked ubiquitin chains on stimulator of interferon genes (STING), suppressing downstream TANK-binding kinase 1 (TBK1)–interferon regulatory factor 3 signaling (IRF3) pathway activation and consequently reducing the production of type I interferons [51].

#### 3.4.2. Role and Molecular Mechanisms of USP21 in CCA

In their 2024 study, Liu et al. elucidated the molecular mechanisms and signaling pathways of USP21 in CCA metabolism [52]. To further investigate the clinical significance of USP21 in CCA progression, they performed immunohistochemical (IHC) staining on a CCA tissue microarray (TMA). Subsequent correlation analysis between USP21 protein expression and clinicopathological characteristics of CCA patients revealed significant associations with tumor location and neural invasion. Univariate analysis demonstrated that USP21 expression, patient sex, tumor size, tumor differentiation, N stage, and surgical margin were significantly correlated with overall survival (OS) in CCA patients. Multivariate Cox regression analysis further identified USP21 expression, tumor size, and N stage as independent prognostic factors for postoperative OS.

Kaplan–Meier survival curves indicated that patients with high USP21 expression had significantly worse OS (*p* = 0.0014) and disease-free survival (DFS, *p* = 0.0342) compared to those with low expression. Collectively, these findings suggest that USP21 serves as a robust predictive biomarker for CCA progression and postoperative patient survival.

**Figure 8:**
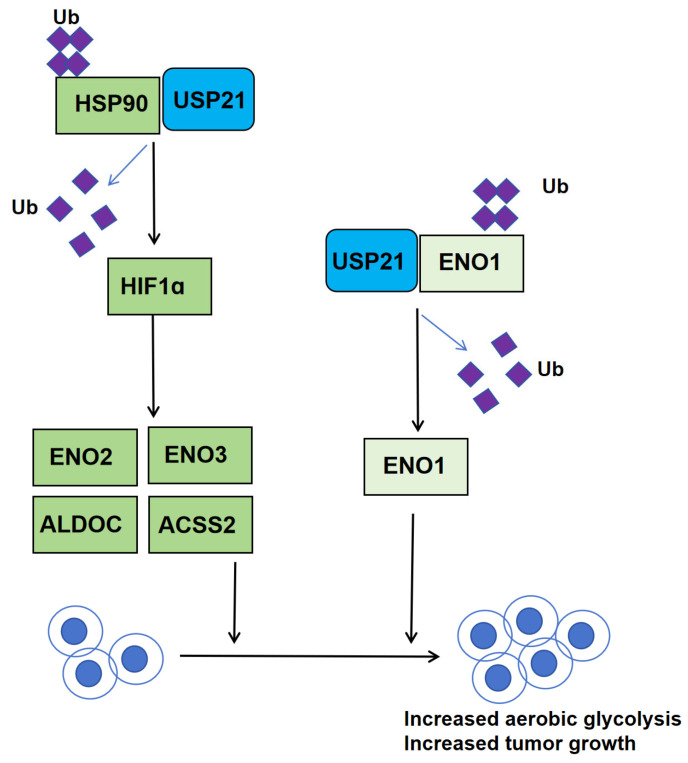
USP21-driven metabolic reprogramming in CCA. USP21 deubiquitinates and stabilizes both HSP90 and ENO1. Stabilized HSP90 preserves HIF1α, which transcriptionally upregulates glycolytic enzymes (ENO2/3, ALDOC, and ACSS2). Simultaneously, stabilized ENO1 directly accelerates glycolytic flux, thereby promoting CCA proliferation.

Mechanistically, USP21 directly interacts with and deubiquitinates HSP90 and ENO1, preventing their K48-linked polyubiquitination and proteasomal degradation(Figure 8). Stabilized heat shock protein 90 (HSP90) maintains hypoxia-inducible factor 1-alpha (HIF1α) protein levels, which in turn transcriptionally upregulates the glycolytic enzymes enolase 2 (ENO2), enolase 3 (ENO3), aldolase c (ALDOC), and acetyl-CoA synthetase 2 (ACSS2) to enhance aerobic glycolysis, while direct stabilization of ENO1 by USP21 further amplifies glycolytic flux and fuels tumor cell proliferation (Figure 8).

Both in vitro and in vivo experiments show that USP21 knockdown suppresses CCA cell proliferation and decreases glucose consumption, lactate production, and ATP levels, whereas USP21 overexpression yields the opposite effects. Pharmacological inhibition of HSP90 with 17-AAG or genetic silencing of ENO1 reverses USP21-mediated glycolytic activation and proliferative advantage, confirming the critical roles of the USP21–HSP90–HIF1α and USP21–ENO1 axes in CCA progression.

Clinically, simultaneous high expression of USP21, HSP90, and HIF1α identifies patients with the poorest outcomes, indicating that combined assessment of these molecules refines risk stratification. Moreover, USP21 overexpression significantly increases resistance to the first-line chemotherapeutic gemcitabine, whereas USP21 inhibition restores drug sensitivity, offering a potential strategy to overcome chemoresistance.

Collectively, this study uncovers a dual mechanism by which USP21 orchestrates aerobic glycolysis and proliferation in CCA, revealing its novel role in mediating chemotherapy resistance. Targeting the USP21/HSP90/HIF1α and USP21/ENO1 signaling axes may provide a rational basis for precision therapies and improved prognostication in cholangiocarcinoma.

### 3.5. USP22

#### 3.5.1. Molecular Characteristics and Molecular Functions of USP22

**Figure 9:**
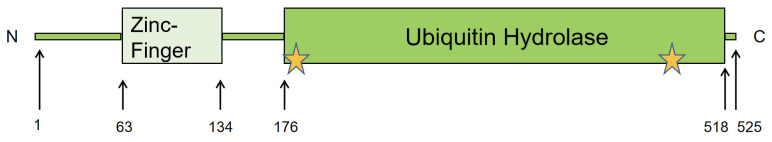
Linear schematic of USP22. Indicated are the catalytic triad (Cys-His-Asp), zinc-binding motifs, and the S1′ pocket that confers selectivity for K27- and K29-linked ubiquitin chains.

USP22 is a deubiquitinase belonging to the USP family, characterized by a conserved catalytic domain adopting the classic “hand-like” fold composed of thumb, palm, and fingers subdomains. Its catalytic triad (Cys-His-Asp) resides within the palm subdomain, while unique structural elements, including zinc-binding motifs and substrate-binding loops, confer specificity toward diverse ubiquitin chain linkages (Figure 9) [53,54]. Notably, USP22 contains an extended N-terminal region that mediates protein–protein interactions and cellular localization, distinguishing it from other USP members.

Functionally, USP22 regulates critical cellular processes through substrate-specific deubiquitination. As a core component of the SAGA (Spt-Ada-Gcn5-acetyltransferase) transcriptional coactivator complex, it stabilizes histone H2B monoubiquitination to modulate chromatin remodeling and gene expression. Beyond epigenetic regulation, USP22 controls cell cycle progression by deubiquitinating cyclin B1 and transcriptional regulators such as FBP1, linking its activity to proliferation and oncogenesis. Recent studies also implicate USP22 in metabolic reprogramming through stabilization of HIF-1α and c-Myc under hypoxic conditions.

The enzyme exhibits remarkable linkage selectivity, preferentially cleaving K27- and K29-linked polyubiquitin chains over other types (K48/K63). This specificity arises from distinct ubiquitin-binding surfaces surrounding its catalytic cleft, including a unique S1’ pocket that accommodates proximal ubiquitin moieties. Structural analyses reveal that USP22’s activity is further modulated by phosphorylation and interacting proteins, suggesting multilayered regulatory mechanisms. These features make USP22 an attractive target for cancer therapy, with several small-molecule inhibitors currently under investigation.

#### 3.5.2. Role and Molecular Mechanisms of USP22 in CCA

In 2021, Tian and colleagues elucidated the critical role of USP22 in promoting cholangiocarcinoma invasion and identified its associated molecular mechanisms [55]. Integrated analysis of 57 paired iCCA tissues via qPCR, IHC, and TCGA data revealed significant USP22 overexpression in tumors, which independently correlated with tumor size > 5 cm, microvascular invasion, lymph node metastasis, and poor prognosis, suggesting its value as a prognostic biomarker. In vitro studies demonstrated that USP22 overexpression markedly enhanced proliferation, migration, and invasion capacities in CCA cell lines (RBE, HCCC-9810), accompanied by EMT induction (E-cadherin downregulation/vimentin upregulation), whereas USP22 knockdown produced opposing effects, validating its oncogenic role.

**Figure 10:**
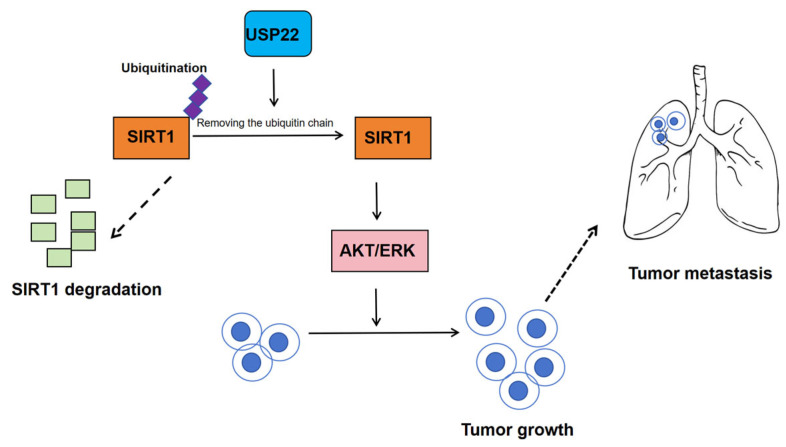
The USP22–SIRT1 oncogenic circuit in CCA. USP22 deubiquitinates and stabilizes SIRT1, thereby activating TAK1/AKT–ERK signaling, inducing the epithelial–mesenchymal transition (EMT), and enhancing invasion and metastasis. Genetic deletion of SIRT1 or pharmacological inhibition of USP22 reverses these phenotypes.

Mechanistically, USP22 stabilizes SIRT1 via deubiquitination, subsequently activating the TAK1/Akt-ERK signaling axis to drive tumor progression. Rescue experiments confirmed that SIRT1 reconstitution fully reversed USP22 knockdown-induced phenotypic defects (Figure 10). In vivo studies further showed USP22 overexpression significantly increased subcutaneous tumor growth and pulmonary metastases in nude mice, effects abrogated by SIRT1 knockout. These findings systematically elucidate the USP22–SIRT1 axis as a central regulator of iCCA progression, providing a rationale for developing targeted therapies against this pathway.

This study makes several original breakthroughs in iCCA research with significant scientific and clinical implications: (1) they are the first to demonstrate USP22’s oncogenic role in iCCA through comprehensive clinical cohort analysis, functional assays, and in vivo models, establishing it as an independent adverse prognostic factor, in contrast to its controversial functions reported in other cancers; (2) the authors discovered a novel USP22–SIRT1 epigenetic axis where USP22 stabilizes SIRT1 via deubiquitination, triggering a previously unreported TAK1/Akt-ERK signaling cascade that bridges tumor metabolism, epigenetics, and signal transduction; (3) the rescue experiments revealed a synthetic-lethal interaction between USP22 and SIRT1, suggesting synergistic therapeutic potential through combined targeting; (4) clinically, the author established a USP22/SIRT1 biomarker system that correlates with tumor invasiveness and enables prognostic stratification; and (5) by validating USP22 as a druggable target and elucidating its SIRT1-mediated deacetylation mechanism, the researchers provide crucial biological rationale and preclinical evidence to accelerate development of USP22 inhibitors or SIRT1 modulators for clinical translation (Figure 10).

## 4. USPs and GBC

### 4.1. USP3

#### 4.1.1. Molecular Characteristics and Molecular Functions of USP3

**Figure 11:**
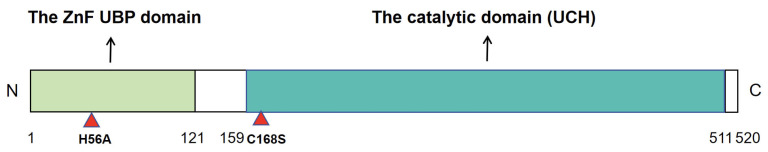
Schematic representation of USP3 domains. Linear domain map: ZnF-UBP (aa 1–121) bearing the ubiquitin C-terminal–binding cleft, and UCH (aa 159–511) containing the catalytic cysteine C168. Inactivating mutations C56A (ZnF-UBP) and C168S (UCH) are indicated.

Ubiquitin-specific protease 3 (USP3) is a cysteine protease and a key member of the deubiquitinating enzyme (DUB) family, distinguished by its unique ability to cleave proline-linked ubiquitin chains. Located on human chromosome 15q22.31, this 520-amino acid protein features two functionally essential domains: (1) a catalytic domain responsible for its protease activity, and (2) a zinc-finger ubiquitin-binding domain (ZnF-UBP) that mediates substrate recognition (Figure 11) [56,57]. Structurally, USP3’s N-terminal region (residues 1–121) forms the ZnF-UBP domain, while the ubiquitin C-terminal hydrolase (UCH) domain (residues 159–511) occupies the C-terminal portion (Figure 11). The ZnF-UBP domain adopts a novel globular fold featuring a deep cleft with a specialized binding pocket for ubiquitin’s C-terminus, a structural architecture unique among known protein domains. Functional studies demonstrate that both domains are indispensable for USP3 activity. The ZnF-UBP domain is impaired by the 56A mutation, while the C168S substitution abolishes catalytic activity in the UCH domain [58,59]. These findings underscore the cooperative mechanism by which USP3 engages and processes ubiquitinated substrates: the ZnF-UBP domain mediates substrate binding, while the catalytic domain executes deubiquitination.

USP3 is a cysteine protease that plays a pivotal role in diverse physiological processes, and its dysregulation is associated with pathological consequences. Functionally, USP3 contributes to DNA repair, cell cycle progression, and apoptotic regulation, with emerging evidence highlighting its critical involvement in genome stability maintenance and inflammatory modulation [60]. USP3 exhibits a distinct bidirectional dysregulation pattern in human diseases. In solid tumors, USP3 is significantly upregulated in gastric cancer, glioblastoma, and breast cancer, where it stabilizes oncoproteins (e.g., c-Myc, MDM2) to promote tumor proliferation, metastasis, and therapy resistance. Conversely, its expression is frequently lost or downregulated in colorectal cancer and acute myeloid leukemia, leading to diminished tumor-suppressive functions that enhance metastatic potential and block cellular differentiation [60]. Notably, USP3’s pathological role extends beyond oncology. It critically regulates cartilage degeneration in osteoarthritis and modulates host responses to HIV-1 and influenza infections [56,57,58,59,60]. Mechanistically, USP3 dually regulates inflammatory pathways by potentiating NF-κB signaling while suppressing type I interferon production. This unique biphasic regulatory property establishes USP3 as both a promising prognostic biomarker and a precision therapeutic target across multiple disease states.

#### 4.1.2. Role and Molecular Mechanisms of USP3 in GBC

In 2022, Liang et al. investigated the effects of USP3 on the GBC progression and glycolysis [61]. They showed that in GBC tissues and cell lines, USP3 is markedly upregulated and positively correlates with the protein level of the glycolytic enzyme pyruvate kinase L/R (PKLR). Functional assays show that USP3 knockdown suppresses cell proliferation, colony formation, and cell-cycle progression, whereas USP3 overexpression reverses these phenotypes and significantly accelerates tumor growth in nude-mouse xenografts. Mechanistically, USP3 removes K48- and K63-linked polyubiquitin chains from PKLR, thereby blocking its proteasomal degradation and stabilizing the protein. Elevated PKLR in turn enhances glucose consumption, ATP, and lactate and pyruvate production, driving the Warburg effect in GBC cells. Rescue experiments confirm that PKLR knockdown reverses the proliferative and glycolytic enhancement induced by USP3 overexpression, establishing a complete “USP3 → PKLR → metabolic reprogramming → tumor progression” signaling axis and providing new prognostic biomarkers and therapeutic targets for GBC.

Liang et al. demonstrated in Biology Direct that the USP3–dynamin 1-like (DNM1L) axis regulates mitochondrial dynamics and promotes liver metastasis in GBC [62]. This study is the first to demonstrate that DNM1L is highly expressed in GBC tissues and cells and is directly stabilized by USP3 via deubiquitination. USP3 binds the GTPase domain (1–302 aa) of DNM1L and selectively cleaves K48-linked ubiquitin chains, prolonging DNM1L half-life and promoting its induction of excessive mitochondrial fission, ATP depletion, reactive oxygen species (ROS) accumulation, and mitochondrial DNA (mtDNA) loss. DNM1L overexpression markedly enhances GBC cell proliferation, migration, and invasion while inhibiting apoptosis; conversely, silencing either DNM1L or USP3 suppresses these malignant phenotypes. In vivo, DNM1L overexpression increases subcutaneous tumor volume by −50% and markedly elevates hepatic metastatic nodules, whereas DNM1L knockdown reduces metastatic foci by at least half, identifying DNM1L as a key driver of GBC liver metastasis.

**Figure 12:**
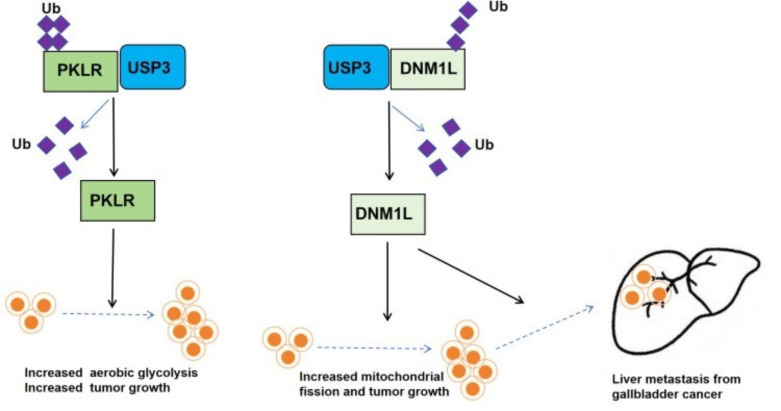
Substrates and mechanism of USP3 in GBC progression. Left: USP3 deubiquitinates PKLR, enhancing glycolytic flux and tumor-cell proliferation. Right: USP3 cleaves K48-linked ubiquitin chains from DNM1L, promoting mitochondrial fission, ROS accumulation, and hepatic metastasis.

To elucidate the oncogenic network governed by DNM1L, integrated transcriptomic and metabolomic analyses were performed. Transcriptomics revealed 2774 differentially expressed genes enriched in DNA replication, cell cycle, and p53 signaling, while metabolomics identified 31 differential metabolites primarily involved in amino-acid, fatty-acid, and nucleotide metabolism. Glutamate was significantly downregulated and proposed as a central metabolic hub controlled by DNM1L. Joint analysis showed that DNM1L drives GBC malignancy and liver metastasis by disrupting the glutamate–pyrimidine–glycine–serine–threonine metabolic axis in concert with mitochondrial dysfunction. Collectively, USP3 stabilizes DNM1L through deubiquitination, thereby remodeling mitochondrial dynamics and cellular metabolism, providing a theoretical basis and experimental foundation for precision therapies targeting the USP3–DNM1L axis in GBC (Figure 12).

## 5. Strategies for Targeting USP1, USP3, USP8, USP9X, USP21 and USP22

Given the well-documented oncogenic or tumor-suppressive roles of individual USP enzymes in BTC, their selective pharmacological modulation represents a rational strategy to complement existing therapies. Below, we outline six viable targeting approaches, the preclinical evidence supporting them, and key translational considerations for each USP.

### 5.1. Targeting the GCN5–USP1–PARP1 Axis in CCA

Zhang et al. (2023) first delineated the pro-oncogenic role of the GCN5–USP1–PARP1 axis in CCA [31]. The acetyltransferase GCN5 acetylates USP1 at Lys130, markedly enhancing USP1’s affinity for PARP1. Acetylated USP1 then deubiquitinates PARP1 at Lys197, preventing its proteasomal degradation and leading to PARP1 accumulation. Elevated PARP1 reinforces DNA-damage repair, thereby promoting CCA cell proliferation, invasion, and metastasis. Analysis of clinical cohorts shows a positive correlation between USP1 and PARP1 expression, and high levels of either protein predict poorer overall survival. This study shows that USP1 stabilizes PARP1 via K197 deubiquitination, fueling cholangiocarcinoma growth and metastasis. Consequently, USP1 inhibitors (e.g., ML323) can be combined with PARP inhibitors (e.g., olaparib) to accelerate PARP1 degradation while simultaneously blocking its DNA-repair–independent oncogenic functions, thereby overcoming PARP-inhibitor resistance. Given that high co-expression of USP1 and PARP1 predicts poor prognosis in CCA, this dual-target regimen is ideally suited for such patients. By further optimizing USP1 inhibitors, adding GCN5 acetyltransferase blockade, and integrating standard chemotherapy or immunotherapy, the USP1-PARP1 co-targeting strategy could be translated into a precision second-line or maintenance therapy for advanced CCA within the next 5–10 years.

### 5.2. Targeting the USPs–DNM1L Axis in GBC

Liang et al.’s 2025 study demonstrates that USP3 removes K48-linked ubiquitin chains on DNM1L, preventing its degradation and thereby amplifying mitochondrial hyper-fission, mtDNA loss, and glycine–pyrimidine metabolic reprogramming that collectively drive GBC proliferation, invasion, and hepatic metastasis [62]. This mechanistic cascade positions USP3 itself as a druggable node: pharmacologic inhibition can simultaneously blunt mitochondrial dysregulation, DNA synthesis hyperactivity, and metabolic rewiring, while USP3high/DNM1Lhigh tumors serve as a precise biomarker-enriched patient population.

Over the next three to five years, organoid-to-orthotopic-to-metastasis mouse models can be leveraged for rapid in vivo validation and iterative optimization of USP3 allosteric inhibitors or proteolysis targeting chimera (PROTAC) degraders, followed by exploration of sequential combinations with gemcitabine–cisplatin or the glutamine antagonist CB-839. Subsequently, an umbrella clinical trial stratified by USP3/DNM1L co-expression should be initiated in locally advanced or high-relapse-risk patients to evaluate the safety and efficacy of USP3-targeted therapy, using circulating mtDNA copy number and MMP2 activity as early pharmacodynamic endpoints to accelerate precision translation.

### 5.3. USP8 Inhibitors DUB-IN-3 Suppress the Malignant Progression of iCCA by Disrupting the USP8–OGT Axis

In a 2024 study published in Cancer Cell International, Long et al. [44] demonstrated that USP8, via its deubiquitinase activity, specifically cleaves K27- and K48-linked ubiquitin chains from O-GlcNAc transferase (OGT), thereby blocking proteasomal degradation and stabilizing the OGT protein. The authors then systematically evaluated the therapeutic potential and molecular mechanism of the USP8 inhibitor DUB-IN-3 in iCCA. In vitro assays (CCK8, EdU, Transwell, and wound-healing) revealed that DUB-IN-3 markedly suppressed iCCA cell proliferation, colony formation, migration, and invasion. In mouse xenograft models, tumors from the DUB-IN-3–treated group were significantly smaller and lighter than those from the control group, indicating robust tumor growth inhibition. Moreover, the anti-tumor efficacy of DUB-IN-3 combined with pemigatinib surpassed that of pemigatinib alone, suggesting that USP8 inhibition can enhance pemigatinib sensitivity. Mechanistically, DUB-IN-3 binds to the catalytic center of USP8—located within the C-terminal USP domain at the critical residue Cys786—thereby abolishing its deubiquitinase function and preventing removal of ubiquitin chains from substrate proteins such as OGT. Consequently, OGT loses its deubiquitination-mediated protection, undergoes proteasomal degradation, and its protein levels drop markedly. Subsequent studies showed that reduced OGT downregulates global O-GlcNAcylation, ultimately suppressing the oncogenic signaling pathways driven by this modification. USP8 inhibitors suppress the malignant progression of iCCA by disrupting the USP8–OGT axis. In summary, agents such as DUB-IN-3 are thus poised to become novel targeted therapeutics for iCCA, particularly for enhancing the efficacy of drugs like pemigatinib.

### 5.4. The Dual Role of USP9X in Tumorigenesis and Its Therapeutic Potential

USP9X, a key member of the deubiquitinase family, exhibits complex and diverse regulatory functions in tumor development, demonstrating a remarkable dual role—either promoting or suppressing cancer—depending on the tumor type and specific molecular context [46,47,63,64].

On the oncogenic side, USP9X stabilizes critical anti-apoptotic proteins (such as MCL-1 and XIAP) through deubiquitination, thereby enhancing tumor cell survival and contributing to chemotherapy resistance, a mechanism observed in multiple malignancies. However, USP9X also exhibits significant tumor-suppressive effects in certain digestive system cancers. For instance, in pancreatic ductal adenocarcinoma (PDAC), colorectal cancer, and cholangiocarcinoma, USP9X inhibits tumor progression by modulating specific signaling pathways [46]. Notably, in cholangiocarcinoma, USP9X promotes apoptosis by deubiquitinating EGLN3 and regulating the expression of KIF1Bβ, highlighting its crucial role in tumor suppression [47].

These findings suggest that the USP9X–EGLN3 signaling axis represents a potential therapeutic target for cholangiocarcinoma. Activating this pathway may lead to the development of novel anti-tumor strategies, offering more precise treatment options for patients. Further research is needed to elucidate the molecular mechanisms of USP9X in different cancers, maximizing its potential for clinical translation.

### 5.5. Therapeutic Potential of Targeting the USP21–HSP90 Axis in CCA

HSP90, a crucial molecular chaperone, plays a pivotal role in tumorigenesis by stabilizing and activating multiple oncogenic proteins, such as EGFR, AKT, and c-Met. A study by Chen et al. (2024) first demonstrated that USP21 promotes CCA progression by deubiquitinating and stabilizing HSP90 [52]. However, the therapeutic potential of targeting USP21 or HSP90 inhibitors in CCA treatment remains unexplored.

Based on these findings, future research should focus on the following directions: developing highly selective USP21 inhibitors to optimize targeted therapy for CCA; investigating the crosstalk between the USP21–HSP90 axis and immune checkpoints (e.g., PD-1/PD-L1) to explore novel combination immunotherapy strategies; and evaluating the synergistic effects of USP21 inhibitors with HSP90 inhibitors (e.g., Ganetespib) to overcome drug resistance and enhance therapeutic efficacy.

In conclusion, the USP21–HSP90 axis is a promising therapeutic target in cholangiocarcinoma. Interrogating this pathway may yield more precise treatment options, but rigorous translational studies are still required to establish its clinical viability.

### 5.6. Therapeutic Prospects of Targeting the USP22–SIRT1 Signaling Axis in CCA

CCA is a highly malignant tumor with a dismal prognosis. In 2021, Tian et al. first demonstrated that USP22 deubiquitinates and stabilizes SIRT1, thereby activating the Akt/ERK pathway, inducing the epithelial-to-mesenchymal transition (EMT), suppressing apoptosis, and markedly promoting proliferation, invasion, and metastasis of CCA cells [55]. Clinical analyses further show that high USP22 expression independently correlates with tumor size > 5 cm, microvascular invasion, lymph-node metastasis, and shorter overall survival, serving as a robust prognostic biomarker. Targeting the USP22–SIRT1 axis therefore offers a novel precision strategy for CCA: (1) developing small-molecule USP22 inhibitors or shRNA/CRISPR-mediated gene silencing to block SIRT1 stabilization, and (2) combining SIRT1 inhibitors (e.g., EX-527, Selisistat) or epigenetic drugs to achieve synergistic anti-tumor effects in USP22high/SIRT1high patients.

USP22 also regulates normal cell-cycle progression; hence, nanocarrier- or tumor-targeted delivery systems are required to improve the therapeutic index and guard against resistance driven by compensatory activation of other deubiquitinases or epigenetic regulators. Future studies should rigorously evaluate the efficacy and toxicity of USP22 inhibitors alone or in combination with SIRT1 inhibitors in humanized mouse models, while immunohistochemistry or liquid biopsy should be employed to screen USP22^high/SIRT1^high populations for prospective precision clinical trials, thereby accelerating the translation of this axis from bench to bedside.

## 6. Future Prospects

The emerging understanding of USPs in BTC pathogenesis opens several promising yet challenging research directions. Building upon current knowledge, we outline key translational opportunities and unanswered questions that warrant future investigation.

### 6.1. Elucidating Tissue-Specific USP Networks

While differential USP expression patterns (e.g., USP7 upregulation in iCCA vs. USP9X loss in GBC) are increasingly recognized, their anatomical subtype-specific regulatory mechanisms remain unclear. Future studies should decipher how tumor microenvironmental cues—including bile acid composition and stromal interactions—spatially regulate USP activity across iCCA, pCCA, and dCCA subtypes. Complementary to this, systematic validation of subtype-dependent USP substrates through integrated proteomics-based ubiquitome profiling and CRISPR screening approaches will be essential to establish mechanistic links between USP dysregulation and BTC pathogenesis.

### 6.2. The Relationship of USP Family and Tumor Environment

Although the USP family has been reported to play roles in GBC and CCA, its interaction with the tumor microenvironment (TME) remains poorly understood. Current studies have primarily focused on the intrinsic functions of USPs in tumor cells, while their regulatory effects on stromal components, such as cancer-associated fibroblasts (CAFs) and immune cells, are largely unexplored. Future research should prioritize investigating the crosstalk between USPs (e.g., USP3, USP7, or USP22) and key TME elements, particularly their modulation of fibroblast activation and immune cell infiltration. Elucidating these mechanisms may uncover novel therapeutic targets to disrupt the protumorigenic niche in biliary tract cancers.

### 6.3. Mechanistic Exploration of USP-Driven Therapeutic Resistance

While USPs are established mediators of chemotherapy resistance (e.g., USP1-mediated cisplatin tolerance), their contributions to immunotherapy resistance in BTC remain largely unexplored. Critical research gaps that need to be addressed include (1) characterizing how specific USPs regulate the stability of immune checkpoints (e.g., PD-L1, B7-H3) in BTC to identify potential immune evasion mechanisms, and (2) systematically evaluating the therapeutic efficacy of combining USP inhibitors with existing anti-PD1 antibodies or chemotherapy regimens using patient-derived organoid models that recapitulate the TME. These investigations will provide crucial insights into USP-mediated resistance pathways and inform the development of rational combination therapies for BTC.

### 6.4. Translation and Intervention

At the translational and interventional level, future efforts should focus on USP3, USP7, and USP22—druggable deubiquitinases whose crystal structures must first be solved to enable structure-based design of high-affinity small-molecule inhibitors. These inhibitors should then be integrated with targeted protein degradation technologies such as PROTACs, molecular glues, or PROTAC-DUB chimeras to achieve rapid, reversible, and tumor-specific depletion of USP enzymes. Building on this foundation, USP inhibitors should be combined with immune checkpoint blockers, FGFR inhibitors, or PARP inhibitors and evaluated in integrated cholangiocarcinoma models that combine patient-derived organoids, patient-derived xenograft (PDX) tumors, and humanized mice. Such systematic studies will assess synergistic efficacy, resistance-overcoming potential, and the toxicity profile of these combinations, providing a precise translational roadmap for subsequent clinical combination regimens.

### 6.5. Clinical Significance and Precision Medicine

To achieve the clinical translation of USP-targeted therapies, a closed-loop translational system must be established that seamlessly connects bench to bedside and back. First, at the companion-diagnostic level, we will develop a tri-modal assay integrating immunohistochemistry (IHC) for spatial USP expression, activity-based probes for real-time enzymatic activity, and ctDNA liquid biopsy for genomic USP alterations, thereby creating a dynamic portrait of USP protein abundance, enzyme activity, and mutational status in each BTC patient. On this basis, a new molecular classification—such as the “USP7-high/PTEN-low” subtype—will be defined to enrich the population most likely to benefit. In the clinical development pathway, an adaptive Phase I trial will be conducted in refractory BTC, using pharmacodynamic markers (e.g., substrate ubiquitination levels) to rapidly identify a safe and biologically effective dose window. Subsequently, a biomarker-driven Phase II study will randomize patients according to USP activity strata (USP22-high vs. USP3-low) and prioritize combination regimens with complementary pathway inhibition, exemplified by pairing a USP7 inhibitor with a PARP inhibitor for the HRD subtype, thereby closing the precision-medicine loop.

The USP family represents a dynamic yet underexplored therapeutic axis in BTC. A multi-omics approach—integrating single-cell sequencing, ubiquitinomics, and chemical proteomics—will be essential to unlock their full clinical potential. Collaborative efforts between basic researchers and clinicians are urgently needed to translate these insights into subtype-tailored USP modulation strategies, ultimately improving the dismal prognosis of BTC patients.

## 7. Conclusions

### 7.1. The Relationship Between BTC and Tumor Progression

BTC, encompassing CCA and GBC, remains a formidable challenge in oncology due to its aggressive nature, late diagnosis, and limited therapeutic options. This review synthesizes current knowledge on six USP members—USP1, USP3, USP8, USP9X, USP21, and USP22—and delineates their context-dependent roles across cholangiocarcinoma and GBC subtypes.

Oncogenic versus tumor-suppressive functions are context-specific.USP1, USP3, USP8, USP21, and USP22 predominantly act as oncogenes by stabilizing pro-tumorigenic substrates (PARP1, PKLR, OGT, HSP90/ENO1, and SIRT1, respectively), whereas USP9X exerts tumor-suppressive activity via EGLN3 stabilization, underscoring the necessity for precision medicine strategies that consider both tumor subtype and USP interactome (Table 1).

### 7.2. The Relationship Between USPs and BTC Metabolism

It has been reported in the literature that USP3 and USP21 play important roles in regulating metabolism and its molecular mechanisms within biliary tract tumors [52,62].

In gallbladder cancer, USP3 stabilizes DNM1L by deubiquitination, inhibiting its proteasomal degradation. This leads to excessive mitochondrial fission and dysfunction, which subsequently interferes with mitochondrial DNA synthesis and amino acid (e.g., glutamate) metabolic pathways. This process remodels the tumor metabolic microenvironment, promoting cancer cell proliferation and liver metastasis [62] (Table 2). This study further integrated transcriptomic and metabolomic data, finding that DNM1L overexpression significantly inhibits ATP production, elevates ROS levels, and reduces mtDNA copy number. These findings suggest that the USP3–DNM1L axis drives the malignant phenotype of gallbladder cancer via the “mitochondrial dynamics-metabolic reprogramming-tumor progression” pathway, providing a theoretical basis for therapeutic strategies targeting mitochondrial metabolism.

In cholangiocarcinoma, USP21 enhances the protein stability of HSP90 and ENO1 by removing their K48-linked ubiquitin chains. HSP90, in turn, stabilizes HIF1A, activating the expression of glycolytic enzyme genes (such as ENO2, ENO3, ALDOC, and ACSS2). ENO1 itself also promotes glycolytic flux. Together, they enhance aerobic glycolysis (the Warburg effect), providing energy and biosynthetic precursors for tumor cells, thereby driving tumor growth and enhancing chemoresistance [52] (Table 2). This study also confirmed that high USP21 expression positively correlates with co-expression of HSP90 and HIF1A. Patients with high concurrent expression of all three proteins had significantly shorter overall survival and disease-free survival, indicating that the USP21–HSP90–HIF1A/ENO1 axis is not only involved in metabolic reprogramming but also holds potential prognostic value, offering new molecular markers and intervention targets for the metabolic targeted therapy of cholangiocarcinoma.

Therapeutic strategies that target USPs—ranging from small-molecule inhibitors and PROTAC-mediated degraders to combination regimens with chemotherapy or immunotherapy—hold promise for overcoming drug resistance and improving clinical outcomes. Nevertheless, major challenges persist in mapping tissue-specific USP networks, deciphering their dynamic interactions with the tumor microenvironment, and developing sufficiently selective and bioavailable inhibitors.

## Figures and Tables

**Table 1:** Summary of USPs family genes’ involvement in cholangiocarcinoma.

USP Family Gene	BTC Type	Function	Substrates	Signal Pathway	Reference
USP1	CCA	USP1 deubiquitinates and stabilizes PARP1, thereby promoting CCA growth and lung metastasis.	PARP1	GCN5-USP1-PARP1	[31]
USP3	GBC	USP3 deubiquitinates PKLR, and DNM1L, promotes glycolytic flux, mitochondrial fission, GBC cell proliferation and hepatic metastasis.	PLKR, DNM1L	USP1-PLKR, USP1-DNM1L	[62]
USP8	iCCA	USP8 drives tumor progression in iCCA through OGT stabilization	OGT	USP8-OGT	[44]
USP9X	CCA	USP9X promotes apoptosis in cholangiocarcinoma by deubiquitinating EGLN3, which upregulates KIF1Bβ expression	EGLN3	USP9X-EGLN3-KIF1Bβ	[47]
USP21	CCA	USP21 promotes aerobic glycolysis and proliferation in cholangiocarcinoma by deubiquitinating and stabilizing both HSP90 and ENO1	HSP90, ENO1	USP21-HSP90-HIF1α-ENO2, ENO3, ALDOC, ACSS2 USP21-ENO1	[52]
USP22	CCA	USP22 promotes CCA progression by inducing EMT and stabilizing SIRT1, which cooperates with USP22 to epigenetically modulate malignancy while exacerbating tumor growth via TAK1/Akt deacetylation.	SIRT1	USP22-SRT1-TAK1/AKT–ERK	[55]

**Table 2:** Similarities and differences between USP3 and USP21 in metabolic regulation of biliary tract tumors.

Feature	USP3 (GC)	USP21 (CCA)
Target protein(s)	DNM1L (mitochondrial fission protein)	HSP90, ENO1 (glycolysis-related)
Ubiquitin chain type	K48-linked ubiquitin chains	K48-linked ubiquitin chains
Metabolic Pathways Affected	Mitochondrial dysfunction, amino acid metabolism (e.g., glutamic acid)	Enhanced aerobic glycolysis (Warburg effect)
Main metabolic effects	Decreased mtDNA, disrupted nucleotide and amino acid metabolism	Increased glycolytic flux and energy supply
Functional consequences	Promotes proliferation, migration, and liver metastasis	Promotes proliferation, chemoresistance, and poor prognosis
Clinical significance	Potential therapeutic target involved in metabolic reprogramming and metastasis	Predictive of prognosis and involved in chemoresistance mechanisms

## Data Availability

No new data were created or analyzed in this study. Data sharing is not applicable to this article.

## Abbreviations

The following abbreviations are used in this manuscript:

ACSS2	Acetyl-CoA synthetase 2
ALDOC	Aldolase c
ATP	Adenosine triphosphate
AXIN	Axis inhibition protein
BACE1	Beta-secretase 1
BTC	Biliary tract cancer
CAFs	Cancer-associated fibroblasts
CCA	Cholangiocarcinoma
CHMP1B	Charged multivesicular body protein 1B
dCCA	Distal cholangiocarcinoma
DNM1L	Dynamin 1 like
DUBs	Deubiquitinating enzymes
EGFR	Epidermal growth factor receptor
EGLN3	Prolyl 4-hydroxylase domain protein 3
ENO2	Enolase 2
ENO3	Enolase 3
ESCRT-II	Endosomal sorting complexes required for transport III
FDA	Food and drug administration
GBC	Gallbladder cancer
GCN5	General control of amino-acid synthesis 5-like 2
GPX4	Glutathione peroxidase 4
GST	Glutathione S-transferase
HCC	Hepatocellularcarcinoma
HIF1α	Hypoxia-inducible factor 1-alpha
HSP90	Heat shock protein 90
iCCA	Intrahepatic cholangiocarcinoma
IFN-β	Interferon-beta
IHC	Immunohistochemistry
IRF3	Interferon regulatory factor 3 signaling
ITCH	Itchy E3 ubiquitin protein ligase
IP	Immunoprecipitation
JAMMs	JAMM/MPN^+^metalloproteases
KAT2A	Lysine acetyltransferase 2A
KDM4C	lysine-specific demethylase 4C
KIF1Bβ	Kinesin family member 1B Beta isoform
KPNA2	Karyopherin subunit alpha 2
LATS2	Large tumor suppressor kinase 2
MAPK	Mitogen-activated protein kinase
MCL-1	Myeloid cell leukemia-1
MCPIPs	Monocyte chemotactic protein-induced proteins
MDA5	Melanoma differentiation-associated protein 5
MINDYs	MIU-containing novel DUB family
MIT	Microtubule-interacting and trafficking
mtDNA	Mitochondrial DNA
NF-κB	Nuclear factor kappa-light-chain-enhancer of activated B cells
NLSs	Nuclear localization sequence
OGT	O-GlcNAc transferase
OTUs	Ovarian tumor proteases
UCHs	Ubiquitin C-terminal hydrolases
PARP1	Poly (ADP-ribose) polymerase 1
pCCA	Perihilar cholangiocarcinoma
PD-L1	Programmed death-ligand 1
PDX	Patient-derived xenograft
PI3K/AKT	Phosphatidylinositol 3-kinase/protein kinase B
PKLR	Pyruvate kinase L/R
PPPDEs	Papain-like peptidases of dsRNA viruses and eukaryotes
PROTAC	Proteolysis targeting chimera
RIP1	Receptor-interacting protein 1
ROS	Reactive oxygen species
RPS16	Ribosomal protein S16
SBM	SH3-binding motifs
STING	Stimulator of interferon genes
SIRT1	Sirtuin 1
TBK1	TANK-binding kinase 1
TGF-β	Transforming growth factor-beta
TME	Tumor microenvironment
TNM	Tumor-node-metastasis
USPs	Ubiquitin-specific proteases
USP1	Ubiquitin specific peptidase 1
USP3	Ubiquitin specific peptidase 3
USP8	Ubiquitin specific peptidase 8
USP9X	Ubiquitin specific peptidase 9, X-linked
USP21	Ubiquitin specific peptidase 21
USP22	Ubiquitin specific peptidase 22
Wnt	Wingless-type
ZNF	Zinc finger protein
ZUP1	zinc finger-containing ubiquitin peptidase 1
